# Expression and function of nuclear receptor co-activator 4: evidence of a potential role independent of co-activator activity

**DOI:** 10.1007/s00018-012-1000-y

**Published:** 2012-05-05

**Authors:** Alexandra Kollara, Theodore J. Brown

**Affiliations:** 1 Samuel Lunenfeld Research Institute, Mount Sinai Hospital, 25 Orde Street, 6-1001TB, Toronto, ON M5T 3H7 Canada; 2 Department of Obstetrics and Gynecology, University of Toronto, Toronto, ON M5S 3G5 Canada

**Keywords:** NcoA4, ARA70, Coactivator, Nuclear receptor, Mitotic spindle, Cancer

## Abstract

Nuclear receptor coactivator 4 (NcoA4), also known as androgen receptor-associated protein 70 (ARA70), was initially discovered as a component of Ret-Fused Gene expressed in a subset of papillary thyroid carcinomas. Subsequent studies have established NcoA4 as a coactivator for a variety of nuclear receptors, including peroxisome proliferator activated receptors α and γ, and receptors for steroid hormones, vitamins D and A, thyroid hormone, and aryl hydrocarbons. While human NcoA4 has both LXXLL and FXXLF motifs that mediate p160 coactivator nuclear receptor interactions, this ubiquitously expressed protein lacks clearly defined functional domains. Several studies indicate that NcoA4 localizes predominantly to the cytoplasm and affects ligand-binding specificity of the androgen receptor, which has important implications for androgen-independent prostate cancer. Two NcoA4 variants, which may exert differential activities, have been identified in humans. Recent studies suggest that NcoA4 may play a role in development, carcinogenesis, inflammation, erythrogenesis, and cell cycle progression that may be independent of its role as a receptor coactivator. This review summarizes what is currently known of the structure, expression, regulation, and potential functions of this unique protein in cancerous and non-cancerous pathologies.

## Introduction

Multiple nuclear receptor co-regulatory proteins have been identified since the prototypical nuclear receptor coactivator, Steroid Receptor Coactivator 1 (SRC1/NcoA1), was first introduced [1]. These co-regulatory proteins, which include both coactivators and corepressors, have been shown to play an important role in modulating physiological and pathological functions of steroid hormone and other nuclear receptors. The p160 family of nuclear receptor coactivators, of which NcoA1 is a member, has been best studied; however, recent studies have also implicated roles for non-p160 coactivators in both development and disease progression. Among these is NcoA4, which has recently been suggested to play a role in development, carcinogenesis, inflammation, erythrogenesis, and cell cycle progression. While some of these activities may involve NcoA4 nuclear receptor co-regulator function, new data suggest that NcoA4 may have additional activities independent of this function.

**Fig. 1:**
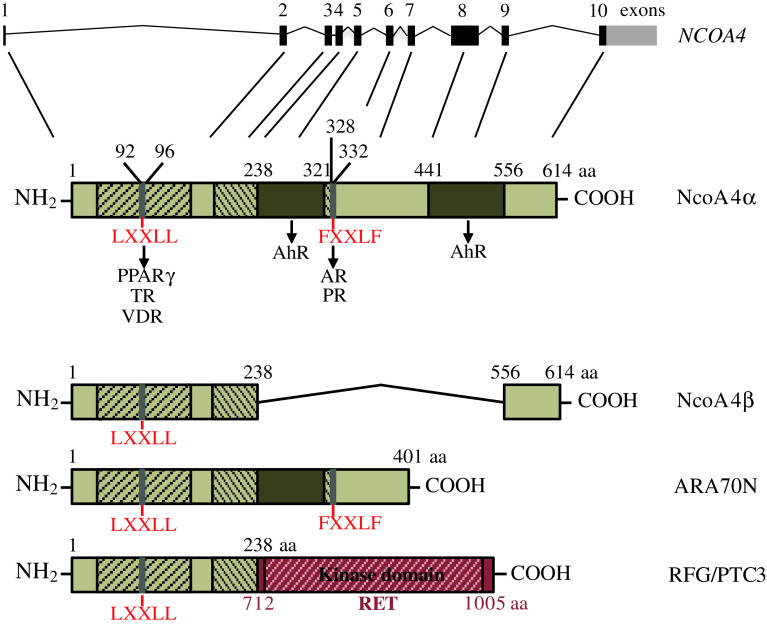
Schematic representation of human NcoA4 variants, ARA70N, and RFG/PTC3. Ensembl Human Genome Browser software indicates the *NCOA4* gene consists of 10 exons. The full-length NcoA4 cDNA encodes a protein of 614 amino acids (NcoA4α). Conserved ARA70 domain I (amino acids 37–167) and domain II (amino acid 138–332) are shown as *hatched areas* and include a FXXLF and LXXLL motif. The FXXLF motif located at amino acids 328–332 is involved in interaction with AR and PR. The LXXLL motif located at amino acids 92–96 is involved in interaction with VDR, TR and PPARγ. Amino acids 231–321 and 441–556 are necessary for optimal AhR coactivation. The shorter NcoA4 variant (NcoA4β) lacks internal amino acids 238–556 of NcoA4α and lacks the FXXLF motif and most of ARA70 domain II. ARA70N consists of the first 401 amino acids of NcoA4α and thus contains both the LXXLL and the FXXLF motifs. Ret Fused Gene (RFG/PTC3) is a fusion protein consisting of the first 238 amino acids of NcoA4 joined to amino acid 712 of the C-terminal region of RET. This fusion protein thus contains the RET tyrosine kinase domain at amino acids 724–1005

NcoA4 was initially discovered as a component of Ret Fused Gene (RFG), a novel fusion protein expressed in papillary thyroid carcinoma, that combines the N-terminal region of NcoA4 with the constitutively active tyrosine kinase domain of the ret oncogene (Fig. 1) [2]. Yeh and Chang [3] first described a function for the intact NcoA4 gene product in 1996. In this study, NcoA4 was identified in a yeast two-hybrid screen as an androgen receptor (AR) interacting protein and was shown to potentiate AR transcriptional activity [3]. Based upon these findings, NcoA4 was referred to as AR-associated protein 70 (ARA70) since it was initially thought to be AR-specific. Subsequent studies, however, indicated that NcoA4 interacted with and regulated the function of additional nuclear receptors including estrogen (ER) [4], progesterone (PR) [5], glucocorticoid (GR) [5], vitamin D (VDR) [6], thyroid hormone (TR) [7], peroxisome proliferator-activated α and γ (PPARαγ) [8, 9], and aryl hydrocarbon (AhR) [10, 11] receptors. This review summarizes what is currently known about the structure, expression, regulation, and potential coactivator and non-coactivator functions of this unique protein.

## Tissue distribution of NcoA4 expression

NcoA4 is ubiquitously expressed in adult mouse tissues including adrenal gland, heart, kidney, lung, intestine, spleen, skeletal muscle, and brain [3, 11, 12]. In contrast, NcoA4 transcripts are not detected in cerebral cortex [3]. There is some disagreement regarding NcoA4 expression in the liver. While Yeh and Chang [3] report a lack of hepatic expression, as determined by northern blot analysis, transcripts and protein have been detected by in situ hybridization, RT-PCR, western blot analysis and immunohistochemistry [11, 12].

NcoA4 protein has also been detected in both female (uterus and ovary) and male (prostate, testis, seminal vesicles, and epididymis) murine reproductive tissues [11, 13]. NcoA4 is highly expressed in mouse ovarian surface epithelial (OSE) cells compared to other ovarian cell types [14]. Gene expression profiling of OSE cells from mice at different reproductive states indicated the highest NcoA4 expression on the evening of pro-estrus compared to either estrus, mid-pregnancy or juvenile stages, suggesting a role of reproductive hormones in regulating its expression. NcoA4 expression has also been detected in murine oocytes [11, 15] and in pre-implantation embryos [16]. A gene expression profiling study comparing the ectoplacental cone region of E7.5 conceptuses to mature E17.5 placentas, showed differential NcoA4 expression, with NcoA4 listed amongst E7.5 stage-specific genes [17]. These studies raise the possibility that NcoA4 may play a role in early embryo and placental development.

A dynamic profile of NcoA4 expression was detected in cardiac, hepatic, and lung tissue during mouse development. Within cardiac tissue, immunostaining was detected from E11.5 to E13.5, whereas a biphasic pattern was detected in hepatic tissue. In lung tissue, the highest NcoA4 expression was detected from E12.5 to E14.5, while staining was undetectable at later stages up to E18.5 [11]. At present, the reasons for the differential expression of NcoA4 at different embryonic stages and tissues are not known. The expression pattern is consistent with a potential contribution to cell proliferation and/or differentiation during development.

Few studies have examined NcoA4 expression in adult or developing human tissues. Gene expression profiling studies indicate human metaphase II oocytes express NcoA4, comparable to what has been reported in mice [18]. Similar studies also indicate NcoA4 expression in the uterine myometrium, with higher levels observed in tissues from pregnant compared to non-pregnant women [19]. Expression of NcoA4 mRNA was also detected by real-time RT-PCR in human luteinized granulosa cells obtained at oocyte retrieval from 198 in vitro fertilization patients [20], and was positively correlated with ERα and aromatase expression. Thus, NcoA4 may contribute to hormonal regulation of follicles.

## Structural properties of NcoA4

The gene encoding NcoA4 is found on chromosome 10q11.2 in humans and 14B in mice. The gene consists of ten exons in both species, resulting in mature transcripts encoding proteins of 614 and 625 amino acids in human and mouse, respectively (http://www.ensembl.org/). Analysis of the NcoA4 amino acid sequence indicates this protein lacks known structural or functional domains with the exception of an N-terminus putative coiled–coil protein–protein interaction domain. Amino acids 37–167 and 198–332 of the human NcoA4 sequences correspond to evolutionarily conserved regions referred to as ARA70 family domains I and II, respectively. Blastp analysis of either the full-length NcoA4 sequence or ARA70-I or -II domains failed to identify related proteins within the human genome, indicating that NcoA4 lacks structurally related family members; however, orthologs exist throughout metazoans [21], suggesting conservation of function.

Two NcoA4 transcript variants have been identified to date in humans: a full-length NcoA4 cDNA that encodes a 614 amino acid protein (NcoA4α) with an estimated molecular weight of 70 kDa [3] and a shorter cDNA possessing an internal 985 base pair deletion encoding a 286 amino acid protein (NcoA4β) with an estimated molecular weight of 35 kDa [22] (Fig. 1). Multiple variants of rodent NcoA4 transcripts have been submitted to the NCBI database; however, their potential translation and function have not yet been explored. In addition to these, we recently discovered a novel murine NcoA4 transcript expressed during early mouse development, consisting of an internal 586 nucleotide deletion corresponding to a lack of exons 4, 5, 6, and 7 and portions of exons 3 and 8 [11]. While expression of protein product for this variant was not detected, a 55-kDa NcoA4 immunoreactive protein was identified that may represent an additional variant. Expression of a shorter uncharacterized NcoA4 transcript variant by northern blot analysis has also been reported in rat testis [22]. The discovery of these rodent variants raises the possibility of additional human variants encoding proteins with diverse functions that could have an important physiological impact.

Most NcoA4-nuclear receptor interactions have been demonstrated using NcoA4α or the N-terminal domain of NcoA4 consisting of amino acids 1–401 (ARA70N; Fig. 1). Human NcoA4α and ARA70N contain two signature nuclear receptor interaction motifs: an LXXLL (LYSLL, where L = leucine, Y = tyrosine and S = serine) motif, initially identified in p160 family members [23], located in the N-terminal region (92–96 amino acids); and an FXXLF (FKLLF, where F = phenylalanine, K = lysine and L = leucine) motif at amino acids 328–332 (Fig. 1) [9, 24]. While the LXXLL motif is involved in the interaction of NcoA4 with PPARγ, VDR, and TR [6, 7, 9], the region involved in the interaction with the ligand binding domain (LBD) of the AR and PR is located within amino acids 321–441, which contains the FXXLF motif [22, 24–27]. Evidence that the FXXLF motif of NcoA4 is essential for NcoA4–AR and NcoA4–PR interactions has been provided by site-directed mutagenesis studies [25, 26, 28]. However, amino acids flanking the FXXLF motif are also important for the functional interaction with AR [26, 29]. While both active androgens, dihydrotestosterone (DHT) and testosterone, promote NcoA4–AR interaction [3, 30], Alen et al. [22] have demonstrated that this interaction also occurs in the absence of exogenous androgen.

Deletion of amino acids 321–441 of NcoA4 diminishes the potentiation of AR transactivation by only 50 % of that achieved with wild-type NcoA4 [24], suggesting that additional regions of NcoA4 contribute to AR–NcoA4 interaction or to NcoA4 coactivator activity. A study by Hu et al. [27] has shown that amino acids 1–175 of NcoA4 are essential for enhancing AR transcriptional activity; however, this does not require the LXXLL motif. It is important to note that this region does not appear to contribute to the physical interaction with AR. Furthermore, inclusion of the C-terminal region of NcoA4 (amino acids 500–614) diminishes NcoA4–AR interaction as compared to ARA70N [24], suggesting the C-terminal region harbors inhibitory sequences.

Studies indicate that NcoA4 interacts with multiple domains of AR, which may involve different regions of NcoA4. Yeast two-hybrid studies have demonstrated that NcoA4 interaction with the AR LBD is further enhanced by the presence of the AR DNA binding domain (DBD) [31, 32]. In addition, maximum induction of AR transcriptional activity by NcoA4 requires its interaction with the N-terminal domain of the AR [24]. Despite lacking the FXXLF motif and its flanking region, NcoA4β interacts with AR in addition to other steroid hormone receptors [22, 33]. However, in contrast to full-length NcoA4, NcoA4β interacts solely with the N-terminal AR domain [33].

Unlike the case with steroid hormone receptors, the ability of NcoA4 to potentiate AhR signaling does not appear to involve either the LXXLL or the FXXLF motif or flanking amino acids 321–441 [10]. Rather, regions within amino acid sequences 238–321 and 441–566 are most likely responsible for optimal AhR transactivation. This is further supported by diminished enhancement of AhR transcriptional activity by ARA70N and NcoA4β relative to full-length NcoA4 [10].

The mouse full-length NcoA4 sequence differs notably from the human sequence in the absence of the LXXLL motif. Murine NcoA4 contains an FXXLF motif at amino acids 334–338. Siriett et al. [12] have reported that the N-terminal region (amino acids 1–501) of mouse NcoA4 interacts directly with the LBD of AR. However, full-length mouse NcoA4 was not able to interact with this AR domain, consistent with human NcoA4 [3], which further supports the existence of an inhibitory sequence located in the C-terminal region of NcoA4.

## Function of NcoA4 as an androgen receptor co-regulator

NcoA4 is best known for its activity as an AR coactivator (Table 1). Early studies demonstrated a ligand-dependent interaction and an increase in AR transcriptional activity by at least 10-fold [3, 34]. However, these studies were performed with ARA70N rather than full-length NcoA4, and subsequent studies have established that this N-terminal portion of the protein provides optimal AR interaction and transactivation enhancement [13, 34–37]. Studies with full-length NcoA4 generally report lower levels of AR potentiation, in the range of 2- to 6-fold [5, 22, 24, 38–40]. Interestingly, despite lacking the FXXLF motif and flanking residues, NcoA4β is at least as effective an AR coactivator as NcoA4α [22, 33], likely due to its interaction with the N-terminal AR domain. Species differences in AR potentiation by NcoA4 are also likely; we found only a modest increase in AR transactivation (1.5-fold) by full-length mouse NcoA4 [11].

**Table 1:** Summary of NcoA4 interacting nuclear receptors and the impact of NcoA4 on their transcriptional activity

Interacting receptors	Ligand	AR transcriptional activity	References
AR	DHT/T/R1881	↑	[3, 5, 11, 22, 24, 34, 38–40]
	E_2_	↑	[35, 41, 42]
	DES/17αE_2_/tamoxifen	–	[42]
	Diadzein	↑	[35]
	Adiol	↑	[43]
	Hydroxyflutamide	↑	[44, 45]
	Hydroxyflutamide	–	[46]
Mutated AR: M745I	R1881/E_2_	↑	[38]
M745I	Progesterone	–	[38]
M745I	Dexamethasone	–	[38]
M745I	Hydroxyflutamide	–	[38]
T877A	Hydroxyflutamide	↑	[46]
E231G/K638M/T857A	R1881/E_2_	↑	[41]
ER	E_2_	↑	[4, 3, 40]
PR	Progesterone	↑	[3]
GR	Dexamethasone	↑	[3]
VDR	1,25-vitamin D	↑	[6]
PPARγ	15dJ2	↑	[9]
RXR	9-*cis* retinoic acid	–	[8, 9]
PPARα	WY14,643	↑	[8]
Mutated PPARα:wild type RXR	WY14,643 and 9-*cis* retinoic acid	↑	[8]
PPARα:RXR	WY14,643 and 9-*cis* retinoic acid	↓	[8]
TR	T_3_	↓	[48]
AhR	TCDD	↑	[10, 11]

A unique aspect of NcoA4 activity at the AR is its reported ability to diminish ligand specificity (Table 1). Overexpression of NcoA4 has been shown to enhance AR transactivation induced by ligands other than active androgens, which may have negative implications for the success of hormonal therapies used in the treatment of prostate cancer. These ligands include 17β-estradiol (E_2_) [35, 41, 42], androstene-3β,17β-diol (Adiol) [43], and the phytoestrogen daidzein [35]. Other ER ligands such as diethylstilbestrol (DES), estrone, 17α-estradiol, and tamoxifen fail to induce AR transactivation in the presence of NcoA4 overexpression, indicating that this effect is ligand-specific [42]. It is important to note, however, that these studies were performed using ARA70N and are reliant on cell-based reporter assays. The effect of full-length NcoA4 has only been examined in a single study, which found only a marginal effect on E_2_-induced AR transcriptional activity [38]. To date, no study has addressed whether this effect can be elicited with an endogenous AR target gene.

In addition to ligand specificity, NcoA4 overexpression reverses the antagonistic activity of hydroxyflutamide (Table 1), an AR antagonist widely used in the treatment of prostate cancer. An agonist effect of hydroxyflutamide induced by ARA70N has been demonstrated using promoter gene assays [44, 45], and is abolished by overexpression of dominant-negative NcoA4 [36]. While this finding has important implications for the development of failed anti-androgen treatment, Brooke et al. [46] found that ARA70N enhances agonist activity of hydroxyflutamide only with human prostate cancer LNCaP cell AR, which contains a point mutation (T877A) within the LBD. No effect was observed with wild-type AR, which is in direct contrast to evidence provided by Yeh et al. [45].

The mutation status of the AR has been shown to influence the potentiation by NcoA4 in a site- and ligand-specific manner (Table 1). For example, NcoA4 enhances estrogen, but not low dose androgen, activation of M745I-mutated AR, a mutation associated with Complete Androgen Insensitivity Syndrome [38]. Hypersensitivity of this receptor to estrogen, coupled with its lower affinity for androgens, is thought to result in the lack of induction of male sexual differentiation by this mutated receptor. Gain-of-function mutations in AR occur during prostate cancer progression, and several of these have been recapitulated in mouse models of prostate cancer to examine their significance (as reviewed by [47]). Using the autochthonous transgenic adenocarcinoma of mouse prostate (TRAMP) model, Han et al. [41] identified somatic AR mutations associated with emergence of androgen-independent prostate cancer. Of three mutations examined, ARA70N was able to increase the activity of E231G-mutated AR in response to either androgen or estrogen ligands, whereas AR-associated protein 160 (ARA160), another AR coactivator, enhanced the activity only of androgens [41]. These studies demonstrate that coactivators differentially modulate mutated ARs.

## Function of NcoA4 as a co-regulator of other nuclear receptors

NcoA4 interacts with several members of the thyroid and steroid hormone receptor superfamily including PR, ER, GR, VDR, PPARγ, PPARα, and TR (Table 1) [4, 6, 8, 9, 22, 48, 49]. While only minimal increases in ligand-activated PR, ER, and GR transcriptional activity (less than twofold) by NcoA4 overexpression have been reported [3, 4, 40], higher activity at VDR, PPARγ, and PPARα has been shown [6, 8, 9]. In addition, NcoA4 activity at the PPARα receptor appears to be context-dependent, functioning as a coactivator in the absence of the PPARα heterodimer partner retinoic X receptor (RXR), and as a repressor in the presence of RXR [8]. Repressor activity of NcoA4 has also been reported for TR [48]. The impact of the NcoA4β variant on these receptors has not yet been addressed, with the exception of the ER, where it has been shown to have similar efficacy as NcoA4α [40].

NcoA4 also interacts with both AhR and its heterodimeric partner AhR nuclear translocator (ARNT) in a ligand-independent manner (Table 1). NcoA4α enhances the AhR transcriptional activity by 3.2-fold in the presence of ligand. In contrast to thyroid and steroid hormone receptor family members, ARA70N and NcoA4β variant were less effective than NcoA4α [10]. In addition, full-length murine NcoA4 was shown to be a more potent coactivator for the AhR than for the AR, with overexpression augmenting the transcriptional activity of AhR by 5-fold and AR by only 1.5-fold [11]. Thus, NcoA4 functions as a co-regulator of diverse nuclear receptor transcription regulators with differences in activity between the two human variants.

## Involvement of NcoA4 in nuclear receptor competition

The interaction of NcoA4 with multiple nuclear receptors raises the possibility that its sequestration by one receptor type might alter the activity of other NcoA4 interacting receptors, with this competition representing a form of cross-talk. Studies indicate that AR competes with AhR, VDR, and PPARγ/RXR for NcoA4 availability [6, 9, 10]. NcoA4 may cooperate with other coactivators, such as AR-associated protein 54 (ARA54), ARA160, retinoblastoma (Rb), breast cancer susceptibility gene 1 (BRCA1), supervillin (SV), and four and a half LIM 2 (FHL2), to synergistically or additively induce receptor transcriptional activity. Additive effects of NcoA4 with Rb and ARA54 have been shown in DU145 cells [50, 51]. Synergistic effects of NcoA4 have been reported with BRCA1, SV, and ARA160 [30, 52, 53], suggesting that NcoA4 may act through a unique mechanism. Synergistic activity of NcoA4 has also been found with FHL2 potentiation of the AhR [54].

The interaction between NcoA4 and AR can be modulated by protein kinases. Blockage of PI(3)K/Akt by dominant-negative Akt or LY294002, a specific PI(3)K antagonist, enhances the impact of ARA70N on AR transcriptional activity [55]. These effects were reversed by addition of constitutively active Akt raising the possibility that NcoA4 may be modulated by its phosphorylation status. However, since PI(3)K/Akt also phosphorylates AR, it is not clear from these studies whether the effect might be due to phosphorylation of AR or NcoA4. While several putative phosphorylation sites exist for NcoA4, phosphorylation of this protein has not as yet been addressed.

NcoA4 lacks intrinsic histone acetyltransferase (HAT) activity [22]; however, both human NcoA4 variants interact with p/CAF [p300/cAMP response element binding protein (CREB) binding protein (CBP)-associated factor], which contains intrinsic HAT activity [22, 34]. Thus, it is possible that NcoA4 may increase the association of p/CAF with the receptor complex.

The predominant cytoplasmic localization of NcoA4, even in the presence of receptor ligands [4, 10, 27, 56, 57], suggests that NcoA4 may act differently than the classical p160 steroid receptor coactivators. NcoA4 may be involved in cytoplasmic events that increase the receptor activity by altering the expression, nuclear translocalization, and stability of the receptor [27]. Indeed, the half-life of AR was increased from 0.8 to 2.3 h in the presence of NcoA4, as demonstrated by pulse chase experiments [27]. Additionally, the predominant cytoplasmic localization of NcoA4 raises the possibility of other functional roles independent of a role as a nuclear receptor coactivator.

## Additional functions of NcoA4

Several coactivators have been found to have functions in addition to their role as nuclear receptor transcriptional modulators. An example of this is SRC3, originally referred to as Amplified in Breast Cancer 1. While this coactivator was initially found to enhance ER signaling, further studies have indicated a promoting role in epithelial–mesenchymal transition (EMT) necessary for metastasis and invasion (as reviewed by Lydon and O’Malley [58]). Moreover, a truncated SRC3 isoform acts to bridge the epidermal growth factor receptor to focal adhesion kinase at the plasma membrane, thereby promoting migration of the cancer cells [59]. Several studies indicate additional roles for NcoA4, some of which may be independent of its activity as a nuclear receptor coactivator.

### Role in proliferation, migration and invasion

Effects of NcoA4 on cell proliferation have been reported that are independent of steroid hormone activity [33, 40]. Despite the fact that both NcoA4α and β function as AR coactivators in cell-based reporter assays, full-length NcoA4 suppressed androgen-induced proliferation of AR-expressing prostate cancer cell lines in vitro and in vivo [32, 33]. In contrast, the NcoA4β variant enhanced cell proliferation and colony-forming ability. Similar effects were found in MCF-7 breast cancer cells [40]. While expression of NcoA4α decreased cell proliferation, NcoA4β expression increased cell proliferation and colony formation independent of steroid hormone.

Despite the similar effects of the two variants in breast and prostate cancer cell lines, the underlying mechanisms appear to differ. In LNCaP cells, 57 % of cells transfected with NcoA4β were in the S phase as compared to 16 % of control cells. This increased cell cycle progression was accompanied by increased cyclin A and decreased p27^kip1^ expression with no effect on cyclin B1 or p21 [33]. NcoA4α overexpression in these cells had no effect on cell cycle progression, but was associated with activated caspase 3 and Bax and decreased Bcl-xL, consistent with increased apoptosis [32]. In comparison, overexpression of NcoA4β in MCF7 cells resulted in decreased p27^kip1^ and increased cyclin B1 and skp2 levels [40]. NcoA4α overexpression in these cells had an opposite effect: increased p27^kip1^ and decreased skp2 expression [40]. The expression of genes related to cell survival were not examined.

NcoA4β and NcoA4α have also been reported to affect the motility of LNCaP and MCF7 cells. NcoA4α inhibited, whereas NcoA4β promoted, migration of LNCaP cells through a matrigel barrier in an androgen-independent manner [32, 33]. In MCF7 cells, only NcoA4β affected (increased) migration in a hormone-independent manner [40]. However, in the presence of androgen, both variants inhibited migration, whereas in the presence of estrogen, both variants promoted migration. These studies demonstrate that NcoA4 affects cell migration independently of the presence of steroid hormone, suggesting involvement of non-coactivator NcoA4 activity; however, since these effects were altered by the presence of steroid hormones, a coactivator function cannot be completely excluded.

### Potential role of NcoA4 in cell division

The recently discovered association of NcoA4 with microtubules is consistent with a potential co-chaperone role in facilitating receptor activity. Several hsp90/steroid receptor interacting proteins have been shown to affect transcriptional activity of steroid hormone receptors through their ability to influence subcellular localization or stability of the receptor [60–65]. AR interaction with various immunophilins including FK506 binding proteins has been postulated to modulate its interaction with dynein and transport along microtubules for nuclear import [66].

**Fig. 2:**
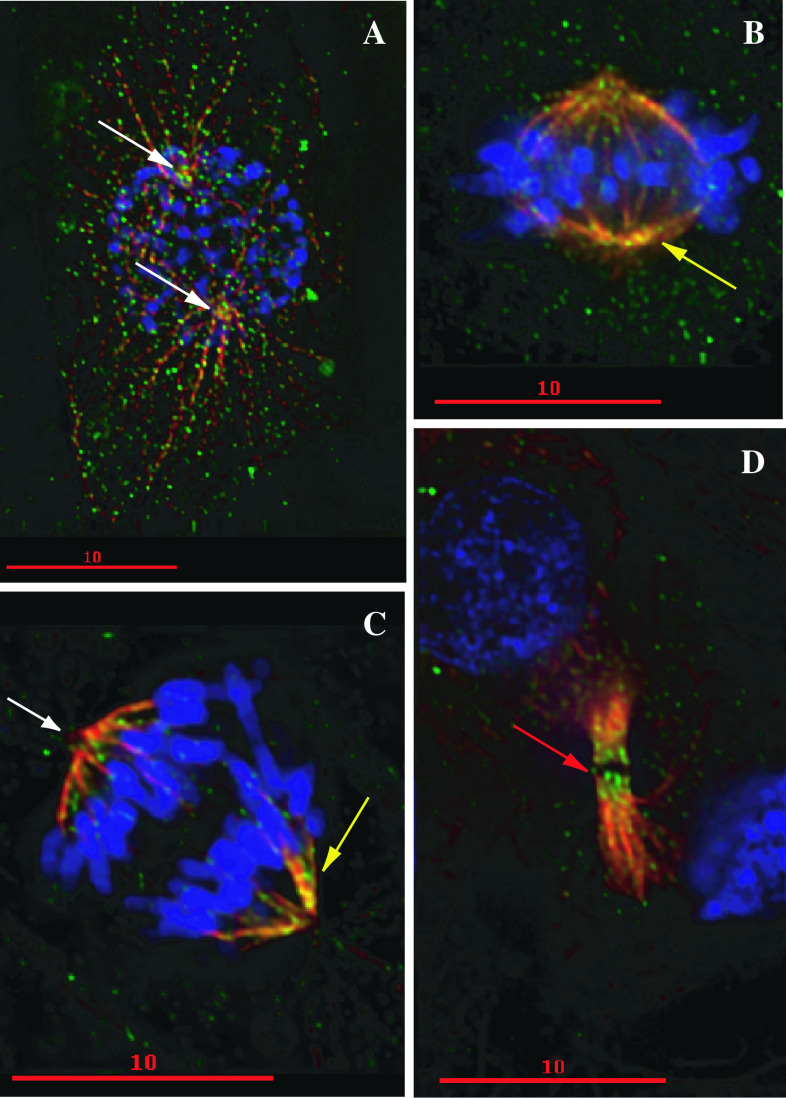
Localization of NcoA4 to the mitotic spindle. Staining of T47D human breast cancer cells for NcoA4 (*green*) and α-tubulin (*red*) was visualized by immunofluorescence and examined by confocal microscopy. Chromatin was visualized by DAPI staining (*blue*). *Yellow* indicates areas of overlapping NcoA4-α-tubulin staining. **a** Prophase cell showing punctate NcoA4 staining associated with microtubules. *White arrows* indicate mitotic organization centers. **b** Metaphase cell showing localization of NcoA4 to mitotic spindles (*yellow arrow*). **c** Anaphase cell showing localization of NcoA4 to mitotic spindles (*yellow arrow*). NcoA4 staining is not observed at the centromere (*white arrow*) at this stage. **d** Telophase cell showing strong NcoA4 staining at the midbodies (*red arrow*). *Bars* 10 μm

NcoA4 has also been found to localize with the mitotic spindle apparatus [21] (Fig. 2), raising the possibility of additional coactivator-independent actions. NcoA4 co-localizes with tubulin and acetylated tubulin at the mitotic spindles during metaphase and anaphase. Dynamic changes in NcoA4 centrosomal distribution, with strong accumulation during interphase and telophase and decreased levels at metaphase and anaphase, indicate that NcoA4 may play an important role in cell division [21]. This, together with NcoA4 staining at midbodies during telophase, is consistent with an action in chromosome segregation and/or cytokinesis. Thus, an intriguing area for further study is a potential role of NcoA4 in cell division and/or the maintenance of genomic stability. Interestingly, NcoA4 was 1 of 30 genes found by suppression subtractive hybridization and differential screening to be downregulated during replicative senescence of 2BS human embryonic lung fibroblasts [67]. Other downregulated genes identified in these non-replicative cells encoded proteins involved with DNA synthesis and RNA processing, cell cycle regulation, cytoskeleton, protein transportation, cell signaling, and metabolism, supporting the possible involvement of NcoA4 in cell division.

## Regulation of NcoA4 expression and function

Few studies have addressed the regulation of NcoA4 expression, and a systematic analysis of the NcoA4 promoter region has yet to be presented. However, regulation by steroid hormones, thyroid hormone, myostatin, and resveratrol has been suggested by several studies. NcoA4 mRNA expression is increased by DHT and estradiol, as well as by tetrahydrogestrinone, an anabolic steroid with potent androgen and progestin effects [68, 69]. Activation of NcoA4 expression by some but not all adipocyte differentiation inducers, including dexamethasone and 3-isobutyl-1-methylxanthine, has been shown in 3T3-L1 preadipocyte cells [70]. These findings suggest regulation of NcoA4 expression by glucocorticoid receptors and by cAMP/cGMP, and raise the possibility that NcoA4 may play a role in early events of adipocyte differentiation.

Upregulation of NcoA4 expression by thyroid hormone has been demonstrated in HepG2 cells transfected either with TRα1 or TRβ1, and in liver from thyroidectomized rats. This regulation appears to be direct as it persists in the presence of cyclohexamide, and TR binding to response elements within the NcoA4 promoter region has been demonstrated by electrophoretic mobility shift assay [48].

Gene expression microarray studies have implicated additional regulators of NcoA4. Among these are sex-determining region Y box 4 (SOX4), a highly conserved transcriptional factor associated with embryonic development and oncogenesis, and manganese superoxide dismutase (SOD2) [71, 72]. Targeted downregulation of SOX4 in LNCaP cells decreases NcoA4 expression whereas downregulation of SOD2 increases NcoA4 expression. The effect of SOD2 targeting siRNA was reversed by N-acetylcysteine treatment, an endogenous antioxidant, indicating that NcoA4 may be regulated by reactive oxygen species. Consistent with this idea, Mitchell et al. [73] has found that the antioxidant resveratrol decreases NcoA4 expression in LNCaP cells.

Murine NcoA4 is negatively regulated by myostatin, a member of the transforming growth factor β superfamily that acts as an inhibitor of skeletal muscle growth [12, 74]. Siriett et al. [12] demonstrated a 1.5-fold increase in NcoA4 transcripts in the biceps femoris muscle of myostatin-null mice compared to wild-type mice. This regulation was verified in C2C12 primary myoblast cells. In the absence of exogenous myostatin treatment, NcoA4 mRNA expression was initiated at 12 h and attained maximum levels at 72 h following differentiation of these cells, suggesting that NcoA4 is involved in late stages of muscle cell differentiation. However, in the presence of myostatin, NcoA4 expression was not detected at any of the time points examined under differentiating conditions [12]. The negative regulation of NcoA4 expression by myostatin, the pronounced skeletal muscle hypertrophy in myostatin-null mice, and the increased expression of AR in muscle hypertrophy [75, 76], are consistent with a coactivator role of NcoA4 in enhancing AR-mediated muscle cell growth.

## Potential involvement of NcoA4 in disease progression

### Thyroid cancer

The involvement of NcoA4 as part of the RET/PTC3 fusion protein in papillary thyroid carcinomas (PTCs) has been studied extensively. RET/PTC rearrangements occur in 10–20 % of PTCs and are associated with radiation exposure and possible induction of chromosomal fragility [77, 78]. The RET/PTC3 chromosomal rearrangement results from the fusion of the constitutively active tyrosine kinase domain of RET with the N-terminal region of NcoA4 (Fig. 1) residing in the long arm of human chromosome 10q11.2 [79, 80]. The ligand-independent dimerization of RET/PTC protein leads to chronic stimulation of MAPK and PI3K-AKT signaling and increased cell proliferation and transformation in thyroid cells [78, 80]. Transgenic mice expressing human RET/PTC3 exclusively in the thyroid develop thyroid hyperplasia and metastatic cancer [81]. RET/PTC3 is able to activate NF-kB and pro-inflammatory mediators in thyroid epithelial cells [82]. While this is known to involve a tyrosine residue within the RET portion of the fusion protein, a potential role of the NcoA4 sequence has not yet been determined.

### Prostate cancer

Single nucleotide polymorphisms (SNP) in chromosome 10q11 have been associated with prostate cancer, with some of these SNPs localizing to NcoA4 [32, 83–85]. However, altered expression of NcoA4 in human prostate cancer has not been firmly established. NcoA4 mRNA and protein expression has been demonstrated in secondary prostate cancer cell lines, LNCaP, PC-3, and DU145 [22, 56, 57, 86–89]. Whereas DU145 and LNCaP cells express only NcoA4α, PC-3 cells also express NcoA4β [56]. Studies examining NcoA4 expression in human prostatic tissues have yielded conflicting results. Li et al. [90] and Ligr et al. [32] reported decreased NcoA4 mRNA expression in both prostate intraepithelial neoplasia, a recognized precursor lesion for prostate cancer, and malignant prostate relative to benign prostate, whereas Mestayer et al. [87] reported similar levels in both normal and malignant prostate. Ligr et al. [32] further showed that NcoA4 overexpression in LNCaP cell xenographs suppressed tumor growth, suggesting a tumor suppressor role of full-length NcoA4. In contrast, Hu et al. [27] demonstrated increased NcoA4 protein levels in neoplastic and malignant prostate compared to benign prostate. Moreover, a more recent study indicates that NcoA4 expression is increased in LNCaP cells maintained in the presence of hydroxyflutamide compared to parental cells [91], suggesting that NcoA4 may play a role in the development of antiandrogen insensitivity.

NcoA4β protein expression has been demonstrated by immunohistochemistry in a subset (15 of 30 cases examined) of prostate cancer cases, with no staining for this variant observed in any cases of benign prostatic epithelium [33]. Differential localization of the two NcoA4 isoforms was noted, with NcoA4α localizing predominantly to the nucleus and NcoA4β to the cytoplasm [89], raising the possibility that the two isoforms may exert different effects.

NcoA4β expression in LNCaP cells has a pronounced effect on gene expression profiles. A genome-wide microarray study identified 953 genes as differentially expressed due to NcoA4β expression [33]. These genes exhibited a greater than 10-fold altered expression level and encode metabolic factors, growth factors, tumor suppressors, oncogenes, transcription factors, and cell adhesion proteins. Genes with the highest fold-increase included HOXA9 (homeobox A9; 160-fold), HOXD13 (homeobox d13; 108-fold), and GR (98-fold), while genes with the highest fold-decrease included NEP (endopeptidase; 1,717-fold), CDH1 (E-cadherin; 1,266-fold), and CLDN3 (Claudin 3; 420-fold). Surprisingly, a greater than 1,000-fold decrease in PSA was also detected in NcoA4β-transfected LNCaP cells. Overall, these findings are consistent with a tumor promoting role of NcoA4β as genes involved in cell adhesion are decreased whereas genes involved with proliferation are increased.

Niu et al. [57] have shown by GST pull-down and co-immunoprecipitation that NcoA4 interacts with PSA and AR, possibly forming a tripartite complex. Co-localization of PSA and NcoA4 was observed in the cytosol of high passage LNCaP cells in the presence of androgen. While this complex was not detected at the promoter of androgen target genes, PSA enhanced AR transcriptional activity in both LNCaP and 22RV1 cells, which was independent of its protease activity and was significantly inhibited by silencing NcoA4 expression. Altogether, these studies indicate the need for further investigations to clarify the expression and/or distribution of NcoA4 isoforms in prostate cancer.

### Breast cancer

The role of ER and PR in breast cancer is well established, whereas the role of AR and AhR is not as well defined. Several studies have investigated the role of steroid hormone receptor coactivators in breast cancer and are suggestive of a role for NcoA4. Decreased NcoA4α mRNA expression in invasive breast cancer relative to benign and in situ carcinoma has been reported, with a greater number of metastatic tumors showing decreased expression [40]. High levels of NcoA4 protein have been shown in human benign breast epithelium and in situ breast carcinomas [56]. Variable protein expression was observed in the invasive carcinomas, with a trend towards decreased NcoA4 protein expression in Her2/neu-positive invasive breast tumors (60 % of Her2/neu-positive vs. 33 % of Her2/neu-negative tumors) [56]. Her2/neu stabilizes AR protein and enhances AR signaling [37, 92, 93], which is generally enhanced by NcoA4 expression. NcoA4 and AR mRNA expression are positively correlated in invasive breast tumors [94]. Since AR is a favorable prognostic indicator, and its action in breast epithelium generally leads to inhibition of cell growth [95–97], loss of NcoA4 and/or AR in the presence of Her2/neu could represent more aggressive subtypes of invasive breast cancer.

Western blot analysis, using protein extracted from invasive ductal carcinomas and adjacent benign breast tissue from four patients, revealed expression of NcoA4α but not NcoA4β in benign tissue, whereas two of the four carcinomas showed markedly decreased expression of NcoA4α and detectable NcoA4β expression [56]. These findings suggest that the two NcoA4 isoforms are differentially expressed in some invasive breast cancers, raising the possibility of a role in disease progression. Further studies are thus required to define the actions of NcoA4 isoforms in breast epithelial cells.

NcoA4 expression has been demonstrated in multiple human breast cancer cell lines with some of these cells expressing NcoA4β [4, 22, 56, 98]. Increased NcoA4 expression was observed in breast cancer cell lines undergoing EMT induced by co-culture with bone marrow-derived mesenchymal stem cells (MSCs) [99]. This study suggests that NcoA4 may reflect or promote breast cancer metastasis. Unfortunately, the microarray approach used did not address whether this effect was isoform-specific.

The effect of NcoA4α and β on mammary gland development was addressed by Wu et al. [40], who generated transgenic mice carrying MMTV-driven transgenes encoding NcoA4α or β. While NcoA4α decreased mammary gland branching, NcoA4β enhanced branching compared to wild-type mice. In addition, mammary hyperplasia was observed in 4-week-old virgin and pregnant NcoA4β mice, but this did not lead to mammary tumor development. These studies demonstrate that NcoA4 modulates breast duct arborization in an isoform-specific manner and plays a role in growth regulation. NcoA4 has also been implicated in mammary cell proliferation by studies performed by Hua et al. [100]. Using a gene-tiling array, they identified NcoA4 as an estrogen-regulated gene and demonstrated ERα binding to the NcoA4 promoter region. Knockdown of NcoA4 expression in MCF7 cells resulted in decreased proliferation under both basal and estrogen-stimulated conditions, consistent with a role of NcoA4 in breast epithelial proliferation.

### Other cancer types

Altered NcoA4 expression has also been demonstrated in other cancers, including ovarian, renal, oral, and colorectal. Using in situ hybridization, we reported upregulated NcoA4 expression in 17 of 20 human invasive ovarian epithelial carcinomas as compared to the non-malignant ovarian surface epithelium [101]. NcoA4 mRNA and protein is also expressed in human ovarian cancer cell lines [86]. As androgen has been implicated in ovarian cancer, these findings raise the possibility that NcoA4 might play a coactivator role in the etiology/progression in a subset of these cancers.

A recent gene expression profiling study identified NcoA4 as one of the genes upregulated in renal cell carcinoma but downregulated in late renal regeneration and repair. Since chronic renal regeneration and repair in individuals with polycystic kidney disease can lead to renal cell carcinoma, these results suggest that NcoA4 expression may be altered in the progression of this cancer [102]. A role of NcoA4 in this progression has not been explored.

A microarray approach has identified NcoA4 transcript levels as one of ten candidate serum markers for oral squamous cell carcinomas (OSCC). An upregulation of NcoA4 RNA in the serum transcriptome of patients with OSCC compared to healthy donors was validated by RT-PCR [103]. While the cell of origin responsible for increased NcoA4 serum transcripts is not known, a possibility is that they are derived from tumor cells undergoing cell death/apoptosis.

In colorectal cancer, studies suggest that NcoA4 may contribute to carcinogenesis associated with loss of epigenetic regulation resulting from impaired histone deacetylase 2 (HDAC2) function and to metastatic progression. Inactivating mutations of HDAC2 have been associated with resistance to histone deacetylase inhibitors in colorectal cancers [104], and Ropero et al. [105] have established that NcoA4 expression is directly repressed by HDAC2. Further studies are required to determine if this increase in NcoA4 is important in the transforming pathway triggered by HDAC2 mutations. Increased NcoA4 expression was also detected in SW620 relative to SW480 colorectal cancer cells [106]. These cell lines were derived from the same patient, with SW480 cells isolated from the primary tumor and SW620 cells isolated from a lymph node metastasis. The increased expression of NcoA4 in the metastatic cell line raises the possibility that NcoA4 may be involved in later progression of the disease or reflect a more aggressive cell line.

### Non-cancerous diseases

Altered NcoA4 expression has been shown in polycystic ovarian syndrome (PCOS) and alopecia, two androgen-associated pathologies. PCOS is associated with oligomenorrhea or amenorrhea, androgen excess, and a high incidence of uterine endometrial hyperplasia. Two studies, which have examined the possibility that NcoA4 expression is altered in the endometrium of PCOS patients, have yielded contradictory results. Quezada et al. [107] reported increased NcoA4 mRNA and protein in endometrial epithelial cells of women with PCOS compared to control women. However, Villavicencio et al. [108] did not observe a difference in NcoA4 protein levels in the epithelium of women with PCOS, PCOS and hyperplasia, hyperplasia only, or normal endometrium. While the reasons for the disparity in these study results is not apparent, in both studies NcoA4 was localized predominantly within the cytoplasm of the epithelial and stromal cells [107, 108]. Possible involvement of NcoA4 in androgenic alopecia is suggested by the finding of decreased NcoA4α expression in the outer hair root sheath of balding areas compared with nonbalding areas, as determined by in situ hybridization [109]. NcoA4β was reduced in the dermal papilla from the balding areas. While the role of NcoA4 in male balding remains speculative, the decrease in NcoA4β associated with a decline in hair follicles is consistent with the likely role of this variant in cell proliferation.

### Erythrogenesis

During development, blood cells of erythroid, myeloid, and lymphoid lineages arise from hematopoietic stem cells, and the regulation of this process by transcription factors is evolutionarily well conserved. Identification of these factors has been facilitated by work with zebrafish mutants. In a whole embryo gene expression analysis of zebrafish mutants exhibiting various hematopoietic deficits, Weber et al. [110] identified NcoA4 as upregulated in erythroid development. This classification of NcoA4 as a potential erythroid transcription-associated factor is supported by its high level of expression in developing human red blood cells. Umbilical cord blood, maternal peripheral blood cells, and adult blood reticulocytes express high levels of NcoA4 [111, 112]. Unverified gene expression profiling studies also indicate that NcoA4 is highly expressed in human platelets [113]. NcoA4 is also expressed by neutrophils and is one of the early genes downregulated by activation of neutrophils by *Escherichia coli* K-12 enterobacteria [114]. This study also revealed expression of a large number of transcriptional regulators, including factors involved with chromatin remodeling, as being rapidly altered upon neutrophil activation; thus, the decrease in NcoA4 may participate in this regulation or reflect the differentiation of these cells.

## Perspectives

NcoA4 is a unique protein that lacks well-defined, known functional domains. The protein is well conserved and paralogs have not been identified. This lack of diversification suggests a conservation of function throughout metazoan evolution [21]. Substantial evidence indicates that NcoA4 is an important nuclear receptor coactivator that undergoes tissue-specific changes in expression during development and in association with certain diseases. Unlike p160 coactivators, NcoA4 has a predominant cytoplasmic localization that does not appear to be altered by treatment with nuclear receptor ligands, suggesting a unique mechanism of action for this coactivator. Although NcoA4 has been widely examined for its action as a coactivator, recent studies show that it associates with cytoskeletal elements and components of the mitotic spindle apparatus. This localization suggests activities for NcoA4 other than that of a nuclear receptor coactivator. Further studies are required to define these potential actions in the context of the evolutionary conservation of this protein.

NcoA4β appears to be emerging as a possible promoter of cell proliferation, migration, and invasion that may be involved in cancer progression and metastasis. Thus, it will be important to delineate the mechanisms underlying differential expression of NcoA4 variants and the impact these isoforms have on non-coactivator functions. Confirmation of the importance of NcoA4 to normal and pathological development is hampered by the lack of a mouse model with targeted disruption of *NcoA4*. Moreover, to our knowledge, NcoA4 has not been the focus of extensive protein–protein interaction studies. Progress in these areas is necessary to reveal novel functions of this protein and to provide a comprehensive understanding of it physiological role.

## References

[1] Katzenellenbogen JA, O’Malley BW, Katzenellenbogen BS (1996). Tripartite steroid hormone receptor pharmacology: interaction with multiple effector sites as a basis for the cell- and promoter-specific action of these hormones. Mol Endocrinol.

[2] Santoro M, Dathan NA, Berlingieri MT, Bongarzone I, Paulin C, Grieco M, Pierotti MA, Vecchio G, Fusco A (1994). Molecular characterization of RET/PTC3; a novel rearranged version of the RET proto-oncogene in a human thyroid papillary carcinoma. Oncogene.

[3] Yeh S, Chang C (1996). Cloning and characterization of a specific coactivator, ARA70, for the androgen receptor in human prostate cells. Proc Natl Acad Sci USA.

[4] Lanzino M, De Amicis F, McPhaul MJ, Marsico S, Panno ML, Ando S (2005). Endogenous coactivator ARA70 interacts with estrogen receptor alpha (ERalpha) and modulates the functional ERalpha/androgen receptor interplay in MCF-7 cells. J Biol Chem.

[5] Gao T, Brantley K, Bolu E, McPhaul MJ (1999). RFG (ARA70, ELE1) interacts with the human androgen receptor in a ligand-dependent fashion, but functions only weakly as a coactivator in cotransfection assays. Mol Endocrinol.

[6] Ting HJ, Bao BY, Hsu CL, Lee YF (2005). Androgen-receptor coregulators mediate the suppressive effect of androgen signals on vitamin D receptor activity. Endocrine.

[7] Moore JM, Galicia SJ, McReynolds AC, Nguyen NH, Scanlan TS, Guy RK (2004). Quantitative proteomics of the thyroid hormone receptor-coregulator interactions. J Biol Chem.

[8] Heinlein CA, Chang C (2003). Induction and repression of peroxisome proliferator-activated receptor alpha transcription by coregulator ARA70. Endocrine.

[9] Heinlein CA, Ting HJ, Yeh S, Chang C (1999). Identification of ARA70 as a ligand-enhanced coactivator for the peroxisome proliferator-activated receptor gamma. J Biol Chem.

[10] Kollara A, Brown TJ (2006). Functional interaction of nuclear receptor coactivator 4 with aryl hydrocarbon receptor. Biochem Biophys Res Commun.

[11] Kollara A, Brown TJ (2010). Variable expression of nuclear receptor coactivator 4 (NcoA4) during mouse embryonic development. J Histochem Cytochem.

[12] Siriett V, Nicholas G, Berry C, Watson T, Hennebry A, Thomas M, Ling N, Sharma M, Kambadur R (2006). Myostatin negatively regulates the expression of the steroid receptor co-factor ARA70. J Cell Physiol.

[13] Thin TH, Kim E, Yeh S, Sampson ER, Chen YT, Collins LL, Basavappa R, Chang C (2002). Mutations in the helix 3 region of the androgen receptor abrogate ARA70 promotion of 17beta-estradiol-induced androgen receptor transactivation. J Biol Chem.

[14] Gava N, Clarke CL, Bye C, Byth K, deFazio A (2008). Global gene expression profiles of ovarian surface epithelial cells in vivo. J Mol Endocrinol.

[15] Stanton JL, Green DP (2001). A set of 840 mouse oocyte genes with well-matched human homologues. Mol Hum Reprod.

[16] Stanton JL, Green DP (2002). A set of 1542 mouse blastocyst and pre-blastocyst genes with well-matched human homologues. Mol Hum Reprod.

[17] Hemberger M, Cross JC, Ropers HH, Lehrach H, Fundele R, Himmelbauer H (2001). UniGene cDNA array-based monitoring of transcriptome changes during mouse placental development. Proc Natl Acad Sci USA.

[18] Grondahl ML, Yding Andersen C, Bogstad J, Nielsen FC, Meinertz H, Borup R (2010). Gene expression profiles of single human mature oocytes in relation to age. Hum Reprod.

[19] Rehman KS, Yin S, Mayhew BA, Word RA, Rainey WE (2003). Human myometrial adaptation to pregnancy: cDNA microarray gene expression profiling of myometrium from non-pregnant and pregnant women. Mol Hum Reprod.

[20] Chang SY, Kang HY, Lan KC, Chang CY, Huang FJ, Tsai MY, Huang KE (2005). Expression of steroid receptors, their cofactors, and aromatase in human luteinized granulosa cells after controlled ovarian hyperstimulation. Fertil Steril.

[21] Kollara A, Ringuette MJ, Brown TJ (2011). Dynamic distribution of nuclear coactivator 4 during mitosis: association with mitotic apparatus and midbodies. PLoS ONE.

[22] Alen P, Claessens F, Schoenmakers E, Swinnen JV, Verhoeven G, Rombauts W, Peeters B (1999). Interaction of the putative androgen receptor-specific coactivator ARA70/ELE1alpha with multiple steroid receptors and identification of an internally deleted ELE1beta isoform. Mol Endocrinol.

[23] Heery DM, Kalkhoven E, Hoare S, Parker MG (1997). A signature motif in transcriptional co-activators mediates binding to nuclear receptors. Nature.

[24] Zhou ZX, He B, Hall SH, Wilson EM, French FS (2002). Domain interactions between coregulator ARA(70) and the androgen receptor (AR). Mol Endocrinol.

[25] Dubbink HJ, Hersmus R, Pike AC, Molier M, Brinkmann AO, Jenster G, Trapman J (2006). Androgen receptor ligand-binding domain interaction and nuclear receptor specificity of FXXLF and LXXLL motifs as determined by L/F swapping. Mol Endocrinol.

[26] Hsu CL, Chen YL, Yeh S, Ting HJ, Hu YC, Lin H, Wang X, Chang C (2003). The use of phage display technique for the isolation of androgen receptor interacting peptides with (F/W)XXL(F/W) and FXXLY new signature motifs. J Biol Chem.

[27] Hu YC, Yeh S, Yeh SD, Sampson ER, Huang J, Li P, Hsu CL, Ting HJ, Lin HK, Wang L, Kim E, Ni J, Chang C (2004). Functional domain and motif analyses of androgen receptor coregulator ARA70 and its differential expression in prostate cancer. J Biol Chem.

[28] van de Wijngaart DJ, van Royen ME, Hersmus R, Pike AC, Houtsmuller AB, Jenster G, Trapman J, Dubbink HJ (2006). Novel FXXFF and FXXMF motifs in androgen receptor cofactors mediate high affinity and specific interactions with the ligand-binding domain. J Biol Chem.

[29] He B, Minges JT, Lee LW, Wilson EM (2002). The FXXLF motif mediates androgen receptor-specific interactions with coregulators. J Biol Chem.

[30] Hsiao PW, Chang C (1999). Isolation and characterization of ARA160 as the first androgen receptor N-terminal-associated coactivator in human prostate cells. J Biol Chem.

[31] Bevan CL, Hoare S, Claessens F, Heery DM, Parker MG (1999). The AF1 and AF2 domains of the androgen receptor interact with distinct regions of SRC1. Mol Cell Biol.

[32] Ligr M, Li Y, Zou X, Daniels G, Melamed J, Peng Y, Wang W, Wang J, Ostrer H, Pagano M, Wang Z, Garabedian MJ, Lee P (2010). Tumor suppressor function of androgen receptor coactivator ARA70alpha in prostate cancer. Am J Pathol.

[33] Peng Y, Li CX, Chen F, Wang Z, Ligr M, Melamed J, Wei J, Gerald W, Pagano M, Garabedian MJ, Lee P (2008). Stimulation of prostate cancer cellular proliferation and invasion by the androgen receptor co-activator ARA70. Am J Pathol.

[34] Yeh S, Kang HY, Miyamoto H, Nishimura K, Chang HC, Ting HJ, Rahman M, Lin HK, Fujimoto N, Hu YC, Mizokami A, Huang KE, Chang C (1999). Differential induction of androgen receptor transactivation by different androgen receptor coactivators in human prostate cancer DU145 cells. Endocrine.

[35] Chen JJ, Chang HC (2007). By modulating androgen receptor coactivators, daidzein may act as a phytoandrogen. Prostate.

[36] Rahman MM, Miyamoto H, Takatera H, Yeh S, Altuwaijri S, Chang C (2003). Reducing the agonist activity of antiandrogens by a dominant-negative androgen receptor coregulator ARA70 in prostate cancer cells. J Biol Chem.

[37] Yeh S, Lin HK, Kang HY, Thin TH, Lin MF, Chang C (1999). From HER2/Neu signal cascade to androgen receptor and its coactivators: a novel pathway by induction of androgen target genes through MAP kinase in prostate cancer cells. Proc Natl Acad Sci USA.

[38] Bonagura TW, Deng M, Brown TR (2007). A naturally occurring mutation in the human androgen receptor of a subject with complete androgen insensitivity confers binding and transactivation by estradiol. Mol Cell Endocrinol.

[39] Kollara A, Brown TJ (2010). Four and a half LIM domain 2 alters the impact of aryl hydrocarbon receptor on androgen receptor transcriptional activity. J Steroid Biochem Mol Biol.

[40] Wu X, Chen F, Sahin A, Albarracin C, Pei Z, Zou X, Singh B, Xu R, Daniels G, Li Y, Wei J, Blake M, Schneider RJ, Cowin P, Lee P (2011). Distinct function of androgen receptor coactivator ARA70alpha and ARA70beta in mammary gland development, and in breast cancer. Breast Cancer Res Treat.

[41] Han G, Foster BA, Mistry S, Buchanan G, Harris JM, Tilley WD, Greenberg NM (2001). Hormone status selects for spontaneous somatic androgen receptor variants that demonstrate specific ligand and cofactor dependent activities in autochthonous prostate cancer. J Biol Chem.

[42] Yeh S, Miyamoto H, Shima H, Chang C (1998). From estrogen to androgen receptor: a new pathway for sex hormones in prostate. Proc Natl Acad Sci USA.

[43] Miyamoto H, Yeh S, Lardy H, Messing E, Chang C (1998). Delta5-androstenediol is a natural hormone with androgenic activity in human prostate cancer cells. Proc Natl Acad Sci USA.

[44] Miyamoto H, Yeh S, Wilding G, Chang C (1998). Promotion of agonist activity of antiandrogens by the androgen receptor coactivator, ARA70, in human prostate cancer DU145 cells. Proc Natl Acad Sci USA.

[45] Yeh S, Miyamoto H, Chang C (1997). Hydroxyflutamide may not always be a pure antiandrogen. Lancet.

[46] Brooke GN, Parker MG, Bevan CL (2008). Mechanisms of androgen receptor activation in advanced prostate cancer: differential co-activator recruitment and gene expression. Oncogene.

[47] Shen MM, Abate-Shen C (2010). Molecular genetics of prostate cancer: new prospects for old challenges. Genes Dev.

[48] Tai PJ, Huang YH, Shih CH, Chen RN, Chen CD, Chen WJ, Wang CS, Lin KH (2007). Direct regulation of androgen receptor-associated protein 70 by thyroid hormone and its receptors. Endocrinology.

[49] Treuter E, Albrektsen T, Johansson L, Leers J, Gustafsson JA (1998). A regulatory role for RIP140 in nuclear receptor activation. Mol Endocrinol.

[50] Kang HY, Yeh S, Fujimoto N, Chang C (1999). Cloning and characterization of human prostate coactivator ARA54, a novel protein that associates with the androgen receptor. J Biol Chem.

[51] Yeh S, Miyamoto H, Nishimura K, Kang H, Ludlow J, Hsiao P, Wang C, Su C, Chang C (1998). Retinoblastoma, a tumor suppressor, is a coactivator for the androgen receptor in human prostate cancer DU145 cells. Biochem Biophys Res Commun.

[52] Ting HJ, Yeh S, Nishimura K, Chang C (2002). Supervillin associates with androgen receptor and modulates its transcriptional activity. Proc Natl Acad Sci USA.

[53] Yeh S, Hu YC, Rahman M, Lin HK, Hsu CL, Ting HJ, Kang HY, Chang C (2000). Increase of androgen-induced cell death and androgen receptor transactivation by BRCA1 in prostate cancer cells. Proc Natl Acad Sci USA.

[54] Kollara A, Brown TJ (2009). Modulation of aryl hydrocarbon receptor activity by four and a half LIM domain 2. Int J Biochem Cell Biol.

[55] Lin HK, Yeh S, Kang HY, Chang C (2001). Akt suppresses androgen-induced apoptosis by phosphorylating and inhibiting androgen receptor. Proc Natl Acad Sci USA.

[56] Kollara A, Kahn HJ, Marks A, Brown TJ (2001). Loss of androgen receptor associated protein 70 (ARA70) expression in a subset of HER2-positive breast cancers. Breast Cancer Res Treat.

[57] Niu Y, Yeh S, Miyamoto H, Li G, Altuwaijri S, Yuan J, Han R, Ma T, Kuo HC, Chang C (2008). Tissue prostate-specific antigen facilitates refractory prostate tumor progression via enhancing ARA70-regulated androgen receptor transactivation. Cancer Res.

[58] Lydon JP, O’Malley BW (2011). Minireview: steroid receptor coactivator-3: a multifarious coregulator in mammary gland metastasis. Endocrinology.

[59] Long W, Yi P, Amazit L, LaMarca HL, Ashcroft F, Kumar R, Mancini MA, Tsai SY, Tsai MJ, O’Malley BW (2010). SRC-3Delta4 mediates the interaction of EGFR with FAK to promote cell migration. Mol Cell.

[60] Czar MJ, Lyons RH, Welsh MJ, Renoir JM, Pratt WB (1995). Evidence that the FK506-binding immunophilin heat shock protein 56 is required for trafficking of the glucocorticoid receptor from the cytoplasm to the nucleus. Mol Endocrinol.

[61] De Leon JT, Iwai A, Feau C, Garcia Y, Balsiger HA, Storer CL, Suro RM, Garza KM, Lee S, Kim YS, Chen Y, Ning YM, Riggs DL, Fletterick RJ, Guy RK, Trepel JB, Neckers LM, Cox MB (2011). Targeting the regulation of androgen receptor signaling by the heat shock protein 90 cochaperone FKBP52 in prostate cancer cells. Proc Natl Acad Sci USA.

[62] Galigniana MD, Erlejman AG, Monte M, Gomez-Sanchez C, Piwien-Pilipuk G (2010). The hsp90-FKBP52 complex links the mineralocorticoid receptor to motor proteins and persists bound to the receptor in early nuclear events. Mol Cell Biol.

[63] Galigniana MD, Radanyi C, Renoir JM, Housley PR, Pratt WB (2001). Evidence that the peptidylprolyl isomerase domain of the hsp90-binding immunophilin FKBP52 is involved in both dynein interaction and glucocorticoid receptor movement to the nucleus. J Biol Chem.

[64] Ni L, Yang CS, Gioeli D, Frierson H, Toft DO, Paschal BM (2010). FKBP51 promotes assembly of the Hsp90 chaperone complex and regulates androgen receptor signaling in prostate cancer cells. Mol Cell Biol.

[65] Wochnik GM, Ruegg J, Abel GA, Schmidt U, Holsboer F, Rein T (2005). FK506-binding proteins 51 and 52 differentially regulate dynein interaction and nuclear translocation of the glucocorticoid receptor in mammalian cells. J Biol Chem.

[66] Buchanan G, Ricciardelli C, Harris JM, Prescott J, Yu ZC, Jia L, Butler LM, Marshall VR, Scher HI, Gerald WL, Coetzee GA, Tilley WD (2007). Control of androgen receptor signaling in prostate cancer by the cochaperone small glutamine rich tetratricopeptide repeat containing protein alpha. Cancer Res.

[67] Zhao L, Tong T, Zhang Z (2005). Expression of the Leo1-like domain of replicative senescence down-regulated Leo1-like (RDL) protein promotes senescence of 2BS fibroblasts. FASEB J.

[68] Labrie F, Luu-The V, Calvo E, Martel C, Cloutier J, Gauthier S, Belleau P, Morissette J, Levesque MH, Labrie C (2005). Tetrahydrogestrinone induces a genomic signature typical of a potent anabolic steroid. J Endocrinol.

[69] Tekur S, Lau KM, Long J, Burnstein K, Ho SM (2001). Expression of RFG/ELE1alpha/ARA70 in normal and malignant prostatic epithelial cell cultures and lines: regulation by methylation and sex steroids. Mol Carcinog.

[70] Nishizuka M, Tsuchiya T, Nishihara T, Imagawa M (2002). Induction of Bach1 and ARA70 gene expression at an early stage of adipocyte differentiation of mouse 3T3-L1 cells. Biochem J.

[71] Liu P, Ramachandran S, Ali Seyed M, Scharer CD, Laycock N, Dalton WB, Williams H, Karanam S, Datta MW, Jaye DL, Moreno CS (2006). Sex-determining region Y box 4 is a transforming oncogene in human prostate cancer cells. Cancer Res.

[72] Sharifi N, Hurt EM, Thomas SB, Farrar WL (2008). Effects of manganese superoxide dismutase silencing on androgen receptor function and gene regulation: implications for castration-resistant prostate cancer. Clin Cancer Res.

[73] Mitchell SH, Zhu W, Young CY (1999). Resveratrol inhibits the expression and function of the androgen receptor in LNCaP prostate cancer cells. Cancer Res.

[74] McPherron AC, Lawler AM, Lee SJ (1997). Regulation of skeletal muscle mass in mice by a new TGF-beta superfamily member. Nature.

[75] Dubois V, Laurent M, Boonen S, Vanderschueren D, Claessens F (2011). Androgens and skeletal muscle: cellular and molecular action mechanisms underlying the anabolic actions. Cell Mol Life Sci.

[76] Inoue K, Yamasaki S, Fushiki T, Okada Y, Sugimoto E (1994). Androgen receptor antagonist suppresses exercise-induced hypertrophy of skeletal muscle. Eur J Appl Physiol Occup Physiol.

[77] Gandhi M, Dillon LW, Pramanik S, Nikiforov YE, Wang YH (2010). DNA breaks at fragile sites generate oncogenic RET/PTC rearrangements in human thyroid cells. Oncogene.

[78] Nikiforov YE, Nikiforova MN (2011). Molecular genetics and diagnosis of thyroid cancer. Nat Rev Endocrinol.

[79] Nikiforov YE (2008). Thyroid carcinoma: molecular pathways and therapeutic targets. Mod Pathol.

[80] Richardson DS, Gujral TS, Peng S, Asa SL, Mulligan LM (2009). Transcript level modulates the inherent oncogenicity of RET/PTC oncoproteins. Cancer Res.

[81] Powell DJ, Russell J, Nibu K, Li G, Rhee E, Liao M, Goldstein M, Keane WM, Santoro M, Fusco A, Rothstein JL (1998). The RET/PTC3 oncogene: metastatic solid-type papillary carcinomas in murine thyroids. Cancer Res.

[82] Russell JP, Engiles JB, Rothstein JL (2004). Proinflammatory mediators and genetic background in oncogene mediated tumor progression. J Immunol.

[83] Chang BL, Cramer SD, Wiklund F, Isaacs SD, Stevens VL, Sun J, Smith S, Pruett K, Romero LM, Wiley KE, Kim ST, Zhu Y, Zhang Z, Hsu FC, Turner AR, Adolfsson J, Liu W, Kim JW, Duggan D, Carpten J, Zheng SL, Rodriguez C, Isaacs WB, Gronberg H, Xu J (2009). Fine mapping association study and functional analysis implicate a SNP in MSMB at 10q11 as a causal variant for prostate cancer risk. Hum Mol Genet.

[84] Pomerantz MM, Shrestha Y, Flavin RJ, Regan MM, Penny KL, Mucci LA, Stampfer MJ, Hunter DJ, Chanock SJ, Schafer EJ, Chan JA, Tabernero J, Baselga J, Richardson AL, Loda M, Oh WK, Kantoff PW, Hahn WC, Freedman ML (2010). Analysis of the 10q11 cancer risk locus implicates MSMB and NCOA4 in human prostate tumorigenesis. PLoS Genet.

[85] Wang Y, Ray AM, Johnson EK, Zuhlke KA, Cooney KA, Lange EM (2010). Evidence for an association between prostate cancer and chromosome 8q24 and 10q11 genetic variants in African American men: The flint men’s health study. Prostate.

[86] Evangelou A, Jindal SK, Brown TJ, Letarte M (2000). Down-regulation of transforming growth factor beta receptors by androgen in ovarian cancer cells. Cancer Res.

[87] Mestayer C, Blanchere M, Jaubert F, Dufour B, Mowszowicz I (2003). Expression of androgen receptor coactivators in normal and cancer prostate tissues and cultured cell lines. Prostate.

[88] Nessler-Menardi C, Jotova I, Culig Z, Eder IE, Putz T, Bartsch G, Klocker H (2000). Expression of androgen receptor coregulatory proteins in prostate cancer and stromal-cell culture models. Prostate.

[89] Peng Y, Chiriboga L, Yee H, Pei Z, Wang Z, Lee P (2008). Androgen receptor coactivator ARA70alpha and ARA70beta isoform-specific antibodies: new tools for studies of expression and immunohistochemical localization. Appl Immunohistochem Mol Morphol.

[90] Li P, Yu X, Ge K, Melamed J, Roeder RG, Wang Z (2002). Heterogeneous expression and functions of androgen receptor co-factors in primary prostate cancer. Am J Pathol.

[91] Wang Y, Li JQ, Shao C, Shi CH, Liu F, Yang ZY, Qiu JX, Li YM, Fu Q, Zhang W, Xue W, Lei YH, Gao JY, Wang JY, Gao XP, Yuan JL, Bao TY, Zhang YT (2011). Androgen receptor coregulators NOCR1, TIF2, and ARA70 may account for the hydroxyflutamide insensitivity of prostate cancer cells. Ir J Med Sci.

[92] Liu Y, Majumder S, McCall W, Sartor CI, Mohler JL, Gregory CW, Earp HS, Whang YE (2005). Inhibition of HER-2/neu kinase impairs androgen receptor recruitment to the androgen responsive enhancer. Cancer Res.

[93] Mellinghoff IK, Vivanco I, Kwon A, Tran C, Wongvipat J, Sawyers CL (2004). HER2/neu kinase-dependent modulation of androgen receptor function through effects on DNA binding and stability. Cancer Cell.

[94] Bieche I, Parfait B, Tozlu S, Lidereau R, Vidaud M (2001). Quantitation of androgen receptor gene expression in sporadic breast tumors by real-time RT-PCR: evidence that MYC is an AR-regulated gene. Carcinogenesis.

[95] Kotsopoulos J, Narod SA (2012). Androgens and breast cancer. Steroids.

[96] Loibl S, Muller BM, von Minckwitz G, Schwabe M, Roller M, Darb-Esfahani S, Ataseven B, du Bois A, Fissler-Eckhoff A, Gerber B, Kulmer U, Alles JU, Mehta K, Denkert C (2011). Androgen receptor expression in primary breast cancer and its predictive and prognostic value in patients treated with neoadjuvant chemotherapy. Breast Cancer Res Treat.

[97] Yu Q, Niu Y, Liu N, Zhang JZ, Liu TJ, Zhang RJ, Wang SL, Ding XM, Xiao XQ (2011). Expression of androgen receptor in breast cancer and its significance as a prognostic factor. Ann Oncol.

[98] Magklara A, Brown TJ, Diamandis EP (2002). Characterization of androgen receptor and nuclear receptor co-regulator expression in human breast cancer cell lines exhibiting differential regulation of kallikreins 2 and 3. Int J Cancer.

[99] Martin FT, Dwyer RM, Kelly J, Khan S, Murphy JM, Curran C, Miller N, Hennessy E, Dockery P, Barry FP, O’Brien T, Kerin MJ (2010). Potential role of mesenchymal stem cells (MSCs) in the breast tumour microenvironment: stimulation of epithelial to mesenchymal transition (EMT). Breast Cancer Res Treat.

[100] Hua S, Kallen CB, Dhar R, Baquero MT, Mason CE, Russell BA, Shah PK, Liu J, Khramtsov A, Tretiakova MS, Krausz TN, Olopade OI, Rimm DL, White KP (2008). Genomic analysis of estrogen cascade reveals histone variant H2A.Z associated with breast cancer progression. Mol Syst Biol.

[101] Shaw PA, Rittenberg PV, Brown TJ (2001). Activation of androgen receptor-associated protein 70 (ARA70) mRNA expression in ovarian cancer. Gynecol Oncol.

[102] Riss J, Khanna C, Koo S, Chandramouli GV, Yang HH, Hu Y, Kleiner DE, Rosenwald A, Schaefer CF, Ben-Sasson SA, Yang L, Powell J, Kane DW, Star RA, Aprelikova O, Bauer K, Vasselli JR, Maranchie JK, Kohn KW, Buetow KH, Linehan WM, Weinstein JN, Lee MP, Klausner RD, Barrett JC (2006). Cancers as wounds that do not heal: differences and similarities between renal regeneration/repair and renal cell carcinoma. Cancer Res.

[103] Li Y, Elashoff D, Oh M, Sinha U, St John MA, Zhou X, Abemayor E, Wong DT (2006). Serum circulating human mRNA profiling and its utility for oral cancer detection. J Clin Oncol.

[104] Ropero S, Fraga MF, Ballestar E, Hamelin R, Yamamoto H, Boix-Chornet M, Caballero R, Alaminos M, Setien F, Paz MF, Herranz M, Palacios J, Arango D, Orntoft TF, Aaltonen LA, Schwartz S, Esteller M (2006). A truncating mutation of HDAC2 in human cancers confers resistance to histone deacetylase inhibition. Nat Genet.

[105] Ropero S, Ballestar E, Alaminos M, Arango D, Schwartz S, Esteller M (2008). Transforming pathways unleashed by a HDAC2 mutation in human cancer. Oncogene.

[106] Futschik M, Jeffs A, Pattison S, Kasabov N, Sullivan M, Merrie A, Reeve A (2002). Gene expression profiling of metastatic and nonmetastatic colorectal cancer cell lines. Genome Lett.

[107] Quezada S, Avellaira C, Johnson MC, Gabler F, Fuentes A, Vega M (2006). Evaluation of steroid receptors, coregulators, and molecules associated with uterine receptivity in secretory endometria from untreated women with polycystic ovary syndrome. Fertil Steril.

[108] Villavicencio A, Bacallao K, Avellaira C, Gabler F, Fuentes A, Vega M (2006). Androgen and estrogen receptors and co-regulators levels in endometria from patients with polycystic ovarian syndrome with and without endometrial hyperplasia. Gynecol Oncol.

[109] Lee P, Zhu CC, Sadick NS, Diwan AH, Zhang PS, Liu JS, Prieto VG (2005). Expression of androgen receptor coactivator ARA70/ELE1 in androgenic alopecia. J Cutan Pathol.

[110] Weber GJ, Choe SE, Dooley KA, Paffett-Lugassy NN, Zhou Y, Zon LI (2005). Mutant-specific gene programs in the zebrafish. Blood.

[111] Goh SH, Josleyn M, Lee YT, Danner RL, Gherman RB, Cam MC, Miller JL (2007). The human reticulocyte transcriptome. Physiol Genomics.

[112] Merkerova M, Vasikova A, Bruchova H, Libalova H, Topinka J, Balascak I, Sram RJ, Brdicka R (2009). Differential gene expression in umbilical cord blood and maternal peripheral blood. Eur J Haematol.

[113] Gnatenko DV, Dunn JJ, McCorkle SR, Weissmann D, Perrotta PL, Bahou WF (2003). Transcript profiling of human platelets using microarray and serial analysis of gene expression. Blood.

[114] Zhang X, Kluger Y, Nakayama Y, Poddar R, Whitney C, DeTora A, Weissman SM, Newburger PE (2004). Gene expression in mature neutrophils: early responses to inflammatory stimuli. J Leukoc Biol.

